# Exploring the underlying mechanisms of exercise as therapy for multiple sclerosis: insights from preclinical studies

**DOI:** 10.3389/fncel.2024.1460262

**Published:** 2024-10-16

**Authors:** Yunpeng Du, Shuhan Dong, Wei Zou

**Affiliations:** ^1^Heilongjiang University of Chinese Medicine, Harbin, China; ^2^First Affiliated Hospital of Heilongjiang University of Chinese Medicine, Harbin, China

**Keywords:** multiple sclerosis, exercise, mechanisms, animal, experimental autoimmune encephalomyelitis sclerosis

## Abstract

Multiple sclerosis (MS) is an immune-mediated disease of the central nervous system CNS characterized by demyelination, inflammation, and neurodegenerative changes, making it the most common nontraumatic disabling neurological disease in young adults. While current pharmacological treatments primarily target immunomodulation or immunosuppression, exercise is gaining increasing attention from the scientific community as an adjunctive therapy. This review explores the potential biological mechanisms of exercise in animal models of MS, focusing on its effects on neuroprotection and inflammation. The review examines how exercise inhibits pro-inflammatory microglial reactivity, stabilizes the blood–brain barrier, and enhances neurotrophic factor expression in animal studies. Future research directions are proposed by summarizing the evidence and limitations of existing animal models of MS, emphasizing the need to further validate these mechanisms in humans to better integrate exercise into the comprehensive management of MS. Additionally, investigating exercise-induced biomarkers for MS symptom reduction may provide a scientific basis for new therapeutic strategies.

## 1 Introduction

Multiple sclerosis (MS) is an acquired inflammatory demyelinating disease of the central nervous system (CNS), characterized by ongoing demyelination, inflammatory, and degenerative affecting the gray and white matter of the brain and spinal cord (Hemmer et al., 2015). Globally, approximately 2.8 million people live with MS, mainly affecting individuals aged 20 to 40, with a higher prevalence in women than in men (Garton et al., 2024). People with MS exhibit a variety of physical and psychological symptoms, such as cognitive and emotional problems, visual disturbances, fatigue, muscle weakness, and movement disorders. Longitudinal studies indicate that the prevalence of MS continues to rise (Koch-Henriksen and Magyari, 2021). The relapsing–remitting type is the most common, with some patients progressing to secondary progressive MS, while 15% exhibit primary progressive MS from onset. Classic MS is prevalent in Europe and the United States, whereas recurrent optic neuromyelitis optica is more common in China.

Immunologic, genetic, and histopathologic findings suggest complex interactions between the immune system, glial cells, and neurons in MS (Kuhlmann et al., 2023). These interactions lead to pathophysiologic changes, including inflammatory demyelination, neuronal damage, and brain lesions. MS treatments often involve immunomodulatory and immunosuppressive drugs to control inflammation and prevent or slow disability progression. However, these approaches have limited efficacy, with 90% of clinical drug trials failing. The high cost and risks associated with clinical trials underscore the need for new therapeutic strategies (Parrilla et al., 2024).

Exercise is increasingly recognized as a non-pharmacological intervention for MS (Motl et al., 2017). Clinical studies have shown that appropriate exercise can not only enhance motor symptoms such as muscle strength and mobility in those with MS, but it can also improve non-motor symptoms such as mood, cognitive impairments, and fatigue (Motl et al., 2017), thereby enhancing quality of life (Farrell et al., 2020). However, support for the benefits of exercise in the literature is inconsistent due to the lack of standardized exercise protocols and heterogeneity in outcome measures (Dalgas et al., 2020). Furthermore, limitations in human studies, such as the difficulty in obtaining histopathologic markers, assessing synaptic function, and exercise-induced neuroplasticity, make a comprehensive analysis of the efficacy of exercise in MS challenging. In particular, most clinical studies have been limited to analyzing markers of peripheral inflammation in advanced stages of the disease and have a brief duration of intervention (Gilio et al., 2022).

Animal studies offer insights by exploring the molecular and cellular effects of exercise. While the potential mechanisms of exercise in MS remain largely unexplored, this review surveys animal model studies related to exercise for MS and analyzes these mechanisms to enhance understanding in this field.

## 2 Method

We conducted a comprehensive search of English-language literature published through July 2024 in PubMed, Web of Science, and Scopus using a specific set of search terms: (exercise) and (rodent or rat or mouse) and (multiple sclerosis or demyelinating disease). We adapted this search strategy to the indexing style of the different electronic databases, and only articles written in English were considered eligible for inclusion. The search covered all fields, including title, abstract, and keywords.

Two reviewers independently screened the titles and abstracts using predefined inclusion criteria focus on the study population (multiple sclerosis animal model), intervention (exercise), and outcome (mechanism). For selected titles, full-text articles were retrieved, and reference lists were searched for additional studies. Principal investigators of the included studies were contacted for further information if necessary. Ultimately, 228 records were identified, and 21 animal studies investigating exercise’s impact on MS were included in the review.

## 3 Result

During full-text evaluation, multiple parameters were collected from 21 articles selected for further analysis and comparison (Jin et al., 2014; Benson et al., 2015; Pryor et al., 2015; Kim and Sung, 2017; Souza et al., 2017; Bernardes and de Oliveira, 2018; Mifflin et al., 2018; Naghibzadeh et al., 2018; Fainstein et al., 2019; Mandolesi et al., 2019; Mifflin et al., 2019; Kim et al., 2020; Shahidi et al., 2020; Chen et al., 2021; Lozinski et al., 2021; Rizzo et al., 2021; Gilio et al., 2022; Kheirdeh et al., 2022; Razi et al., 2022; Sajedi et al., 2023; Banasadegh et al., 2024). The information included were authors, year of publication, animal model, type of exercise, exercise parameters (single time, frequency, total time), location of detection, and mechanistic changes. Our review focuses on rat and mouse models of MS because these were the only models identified in our comprehensive literature search, ensuring reliable and consistent results. All this information was collected in Table 1.

**Table 1:** Effect of exercise on multiple sclerosis and its underlying mechanisms.

Authors	Animal species^*^	Model	Exercise method	Exercise parameter	Site of sample collection	Involved in pathways	Mechanism
Banasadegh et al. (2024)	Female C57BL/6 mice, 6–8 wks old	EAE	Aerobic swimming	30 min, 7d/w, 4wks	Brain	/	BDNF **△**, miR-142-3p **▽**
Sajedi et al. (2023)	Female C57BL/6 rats, 8 wks old	CPZ	Aerobic training	10-20 min, 5d/w, 8wks	Striatal	/	MBP, nestin mRNA, myelination **△**
Kheirdeh et al. (2022)	Female Sprague–Dawley rats, 8–10 wks old	EAE	Aerobic training	25-35 min, 5d/w, 5wks	Hippocampal	/	CB1R **△**
Gilio et al. (2022)	Female C57BL/6 mice, 9wks old	EAE	Running wheel	/	Striatal	/	IL-2 **▽**
Razi et al. (2022)	Female Lewis rats, 6–8 wks old	EAE	Treadmill training	30/40/50/60 min, 6wks	Brain, hippocampal	/	GFAP, Ang-1 proteins neuronal apoptosis **▽** TJ proteins **△**
Rizzo et al. (2021)	Female C57BL/6 mice, 5–6 wks old	EAE	Voluntary exercise	/	Hippocampal	/	TNF, microgliosis, IL-1β **▽**
Chen et al. (2021)	Female C57BL/6 mice, 3–4 wks old	EAE	Strength Exercise	20/40/60 min, 1d/6d/1wks, 4wks;	Serum, fecal	Gut Microbiota	Firmicutes/Bacteroidetes, Th17 **▽** SCFA, mucosal permeability, Treg **△**
Lozinski et al. (2021)	Female C57BL/6 mice, 6–12 wks old	LPC	Running wheel	/	Serum, spinal cords	/	Spinal Cord Proteins: 86 proteins **△** 85 proteins **▽** Serum: 14 proteins **△** 11 proteins**▽**
Kim et al. (2020)	Female Lewis rats, 9 wks old	EAE	Swimming	30 min, 26d.	Spinal cords	/	TNF-α、IL-1β、IL-6、COX-2、iNOS **▽** NGF、MBP、PLP **△**
Shahidi et al. (2020)	Female C57BL/6 mice, 6–8 wks old	EAE	Running wheel/ Swimming	Running Wheel: 60 min, 5d/w, 4wks; Swimming: 30 min, 5d/w, 4wks	Spinal cords	/	Body weight, demyelination, expression of genes **▽**
Fainstein et al. (2019)	Female SJL/JCrHsd mice, 6–7 wks old	EAE	Treadmill training	10/20/30 min, 5d/w, 6wks	Spinal cords	/	1.1.1 TNF α, TGF β, LN-T cell **▽**
Mandolesi et al. (2019)	Female C57BL/6 N mice, 8 wks old	CPZ	Running wheel	3-6wks	Striatal	/	Iba1, new OL **▽**
Mifflin et al. (2019)	Male and female C57BL/6 J mice, 6–8 wks old	EAE	Running wheel	60 min	Splenocyte, dorsal root ganglia	/	Male/Female: IFN-*γ* **△** Male: TNFα **▽** IL-17A, Ca ^2+^ **△** Female: TNF*α* **△**
Bernardes and de Oliveira (2018)	Female C57BL/6 J mice, 4–6 wks old	EAE	Treadmill training	30 min, 5d/w, 6wks;	Spinal cords	/	Microglial/macrophage response **△**
Mifflin et al. (2018)	Male and female C57BL/6 J mice, 6–8 wks old	EAE	Running wheel	60 min, 4wks	Brain	/	Female: Pregnenolone **△**
Naghibzadeh et al. (2018)	Male C57BL/6 mice, 4wks old	CPZ	Treadmill training	30 min, 5d/w, 5wks	Brain, hippocampal	/	BDNF, GDNF, NGF **△**
Souza et al. (2017)	Female C57BL/6 mice, 6–12 wks old	EAE	Strength training, Endurance training	30 min, 5d/w, 4wks; 10 min, 5d/w, 4wks.	Splenocyte, spinal cords	Nrf2/ARE	IFN-γ, IL-17, IL-1β, IL-6, MCP-1, (TNF)-α **▽** CD25, IL-10 **△**
Kim and Sung (2017)	Female C57BL/6 mice, 10wks old	EAE	Treadmill training	30 min, 5d/w, 4wks	Brain, hippocampal	/	bcl-2, BDNF, MBP, CNPase, brdU, DCX **△** bax, caspase-3, TUNEL **▽**
Benson et al. (2015)	Female C57BL/6 mice, 10-12wks old	EAE	Running wheel	/	Spinal cords	/	CD3+ T-cells, Iba1, COX IV, Tom20 **▽**
Pryor et al. (2015)	Male C57BL/6 J mice, 10wks old	EAE	Running wheel	7d/w, 4wks	Spinal cords	/	CCL20 **▽**
Jin et al. (2014)	Male Sprague–Dawley rats, 30 wks old	EtBr	Swimming	30 min, 14d	Hippocampal	/	TUNEL, caspase-3 **▽**

EAE, experimental autoimmune encephalomyelitis; MBP, Myelin basic protein; ARE, antioxidant response elements; Nrf2, nuclear factor erythroid 2-related factor; IFN, interferon; IL, interleukin; TNF α, tumor necrosis factor α; CPZ, Cuprizone; MBP, Myelin basic protein; LPC, lysophosphatidylcholine; OL, oligodendrocytes; EtBr, ethidium bromide. ^*^ This table exclusively includes studies on rat and mice MS models.

## 4 Mechanisms of exercise on multiple sclerosis

**Figure 1:**
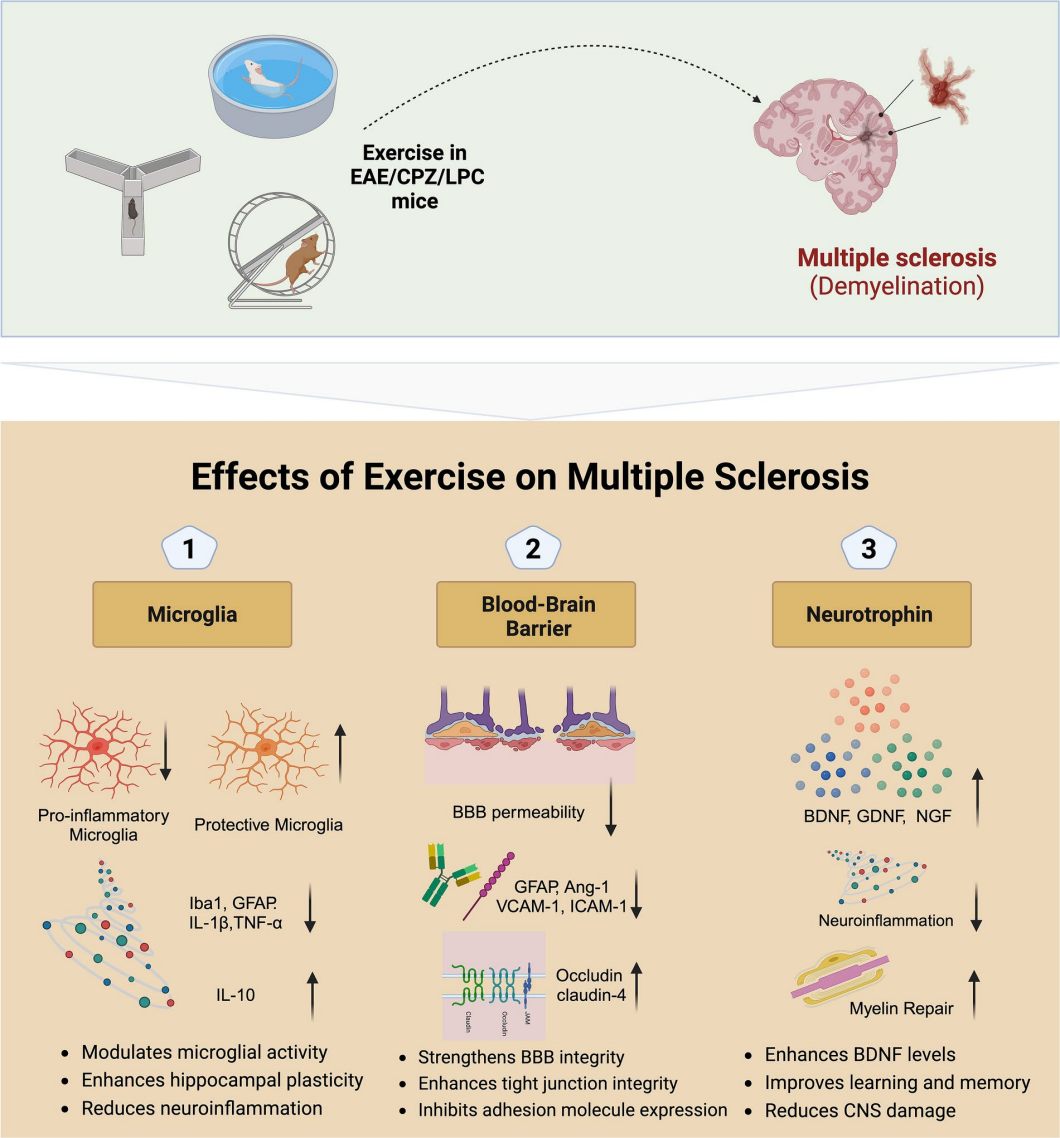
Potential mechanisms of exercise on multiple sclerosis. EAE, Experimental autoimmune encephalomyelitis; CPZ, Cuprizone; LPC, Lysophosphatidylcholine; BBB, Blood–brain barrier; BDNF, Brain-derived neurotrophic factor; GDNF, Glial cell line-derived neurotrophic factor; NGF, Nerve growth factor; GFAP, Glial fibrillary acidic protein; IL, Interleukin; TGF, Transforming growth factor; VCAM, Vascular cell adhesion molecule; ICAM, Intercellular adhesion molecule.

Investigating the mechanisms by which exercise affects MS through randomized controlled trials is challenging. However, animal studies offer a valuable alternative for exploring these biological mechanisms. In this context, we have reviewed preclinical studies on MS animal models, including attenuating the reactivity of pro-inflammatory/neurodegenerative microglia, stabilizing the blood–brain barrier (BBB), and enhancing neurotrophin expression (Figure 1).

### 4.1 Attenuating the reactivity of pro-inflammatory/neurodegenerative microglia

Microglia, the primary immune cells in the CNS, can switch between pro- and anti-inflammatory states. They are implicated in hereditary white matter encephalopathies and neurodegenerative diseases, playing a critical role in MS by contributing to lesion formation and neural repair. During the acute injury phase of MS, approximately 40% of phagocytes are pro-inflammatory M1 microglia, which exacerbate nerve fiber damage and demyelination by releasing inflammatory factors and oxidative substances (Zrzavy et al., 2017). As inflammation progresses, there is a gradual increase in the number of protective M2-type microglia, which can release anti-inflammatory cytokines, such as IL-10, that help to alleviate inflammation and promote tissue repair (Chu et al., 2018; Tikus et al., 2023).

Exercise is thought to promote CNS health by modulating microglia reactivity (Mee-Inta et al., 2019). Inflammation disrupts synaptic plasticity in the CA1 region of the hippocampus and exacerbates neuronal damage through a reduction in GABAergic transmission (Nisticò et al., 2013). Rizzo et al. (2021) verified the protective effects of prolonged voluntary exercise on hippocampal function in a model of experimental autoimmune encephalomyelitis (EAE). Specifically, in terms of enhancing neuroprotection, exercise maintained the neuronal network and synaptic plasticity in the CA1 area by boosting the survival of parvalbumin-positive (PV+) interneurons. In terms of anti-inflammation, exercise significantly reduced microglial hyperplasia and TNF expression in microglia and decreased IL-1β levels in the CA1 region. In addition, in an EAE mouse model, high-intensity continuous training exhibited a significant 61% reduction in the number of neurotoxic Iba1+, iNOS+ (M1 type) microglia compared to sedentary mice (*p* < 0.05) (Zaychik et al., 2021).

Mandolesi et al. (2019) showed that voluntary running-wheel exercise for 6 weeks from the date of disease induction significantly suppressed microgliosis (Iba1), astrogliosis, and demyelination in the corpus callosum and striatum in a mouse model of MS with cuprizone-induced demyelination. These results suggest that exercise aids functional recovery by limiting myelin destruction, preserving myelin-associated proteins, and reducing axonal damage, thereby promoting microglia’s shift to a reparative phenotype, reducing neuroinflammation, and supporting nerve regeneration.

Overall, exercise modulates microglial reactivity and phenotype, significantly reducing MS-related neuroinflammation and neuronal damage. This modulation may also prevent chronic neurodegenerative changes over the long term. Future studies should explore how different types and intensities of exercise influence microglial phenotype transitions and validate these mechanisms across various stages of MS. Understanding these effects more comprehensively could provide a strong scientific basis for developing exercise-based interventions.

### 4.2 Stabilizing the blood–brain barrier

The BBB regulates the entry of molecules, ions, and cells from the blood into the CNS, thereby stabilizing and protecting the neuronal microenvironment. In MS, destruction of the BBB is a critical early step in the autoimmune attack on the CNS, leading to demyelination and axonal loss, ultimately resulting in neurodegeneration and irreversible neurological dysfunction (Zierfuss et al., 2024). Pro-inflammatory cytokines, such as IFN-*γ* and TNF-*α*, stimulate the BBB endothelium to down-regulates tight junctions and up-regulates cell adhesion molecules such as ICAM-1 and VCAM-1, destabilizing the BBB and increasing leukocyte migration into the CNS (Nishihara et al., 2022; Zierfuss et al., 2024; Rasool et al., 2024).

Exercise is thought to counteract BBB hyperpermeability through various mechanisms. A study by Razi and colleagues explored the effects of aerobic training on the integrity of the BBB and neuronal death in an EAE animal model of MS (Razi et al., 2022). The results showed a significant decrease in the expression of glial fibrillary acidic protein (GFAP) and angiopoietin-1 (Ang-1), reduced neuronal apoptosis, and increase tight junction proteins (TJ proteins) expression in the aerobically trained+EAE group compared to the non-exercised EAE group. This suggests that aerobic exercise may reduce GFAP expression by decreasing the release of inflammatory mediators from microglia. The increase in Ang-1 protein leads to an increase in BBB permeability, which may be related to the downregulation of TJ protein and the attraction of immune cells to endothelial cells. Aerobic training attenuates EAE-induced increase in BBB permeability and its associated complications by decreasing Ang-1 protein expression. Overall, aerobic training contributes to the maintenance of BBB integrity through the interplay of these mechanisms, thus providing a potential protective effect in MS models.

Souza et al. (2017) reported that physical exercise, especially a regimen focusing on strength and endurance (30 min per day, five times per week for 4 weeks), effectively inhibited pathology progression in an MS animal model. This exercise inhibits the expression of adhesion molecules through immunomodulatory effects and reestablishes tight junctions in spinal cord tissues, thereby limiting the permeability of the BBB and the migration of autoreactive T cells to the CNS. Specifically, aerobic exercise restores tight junction proteins in the CNS, such as occludin and claudin-4, to baseline levels after EAE induction. These proteins are key components of the structural integrity of the BBB, and they limit the entry of substances into the brain through cellular gaps by means of tight junctions between cells. Schreibelt and coworkers showed that exercise significantly increased the expression of these proteins in the spinal cord of EAE mice, thereby protecting the BBB by enhancing the structural integrity of the tight junctions (Schreibelt et al., 2006).

In addition, exercise inhibited the expression of the adhesion molecule PECAM-1 and improved BBB function by decreasing the expression of intercellular adhesion molecule 1 (ICAM-1) and exocytotic vascular cell adhesion molecule 1 (VCAM-1), which are implicated in inflammation and immune cell infiltration (Souza et al., 2017). Pro-inflammatory cytokines such as IFN-*γ* and TNF-*α* are known to increase BBB permeability by modulating these adhesion molecules. Exercise restores BBB permeability by modulating the expression of these molecules and decreases the expression of inflammatory factors (IFN-γ, IL-17, and IL-1β), effectively attenuating EAE-induced BBB permeability and the ensuing complications. While strength and endurance activities focus differently, both maintain BBB integrity through these mechanisms, offering potential protective effects in treating immune-mediated diseases like MS. Regular aerobic exercise may therefore serve as a key non-pharmacologic intervention for MS treatment and prevention.

In summary, exercise enhances the integrity of the BBB by regulating the expression of inflammatory cytokines and adhesion molecules. This leads to reduced inflammation and limits the infiltration of immune cells into the CNS. These findings underscore the critical role of exercise in protecting neural environments from MS-related damage and highlight its potential as a non-pharmacological strategy for treating and preventing MS. Future studies should further investigate the long-term protective effects of exercise on the BBB and its specific impacts at various stages of MS.

### 4.3 Enhancing neurotrophin expression

Decreased secretion of neurotrophic factors is an important factor contributing to inadequate neuroprotection in neurodegenerative diseases such as MS (Razavi et al., 2015). Among them, key neurotrophic factors such as BDNF, GDNF, NGF, and CNTF play complex and critical roles in neuronal survival, immune regulation, and neuroplasticity. BDNF, in particular, is not only crucial in the growth and survival of neurons and oligodendrocytes, but also plays a role in neurotransmitter regulation and neuroplasticity. Notably, in a mouse model of EAE, the elevation of BDNF in the brain at the peak of the disease may be a self-repair mechanism for neuronal and glial damage (Razavi et al., 2015; JUBAIDI et al., 2024). Subsequent decline in intracerebral BDNF indicates disease damage (Aharoni et al., 2005). BDNF is an important molecular mediator of neuronal survival and is associated with the beneficial effects of exercise on the CNS.

Earlier studies found that EAE mice engaging in regular swimming exercise had significantly higher BDNF levels in the brain and spinal cord, lower inflammatory factors in the brain, and higher inflammatory factors in the spinal cord compared to non-exercising EAE mice (Bernardes et al., 2013). In addition, exercise significantly reduced disease severity and weight loss and slowed the growth of clinical scores in EAE mice, effects that suggest that exercise may influence myelin denaturation and repair processes by improving nerve regeneration and reducing pathologic inflammatory responses that promote the proliferation or regeneration of oligodendrocytes in the lesion area. These findings emphasize the important role of regular exercise in the treatment of EAE and potentially in the treatment of MS-related symptoms. Overall, exercise-induced increases in neurotrophic factor secretion may tilt the balance toward a less neurotoxic environment in which CNS cells can more effectively promote repair and regeneration.

Recent studies corroborate these findings. Four weeks of swimming exercise increased BDNF, decreased miR-142-3p, and improved cognitive performance in an EAE mouse model (Banasadegh et al., 2024). Exercise promotes BDNF secretion by increasing neuronal sensitivity to BDNF, which has a direct effect on key regions of learning and memory. In addition to the EAE mouse model, the cuprizone-induced demyelination model mouse type also showed a potential protective effect of exercise (Naghibzadeh et al., 2018; Begum et al., 2022). Summarizing the two training modes, both high-intensity interval training (HIIT) and low-intensity continuous training (LICT) increased the mRNA levels of BDNF, GDNF, and NGF, with HIIT being more effective (Naghibzadeh et al., 2018). Compared to LICT, HIIT provided stronger neuroprotective effects in the hippocampus by normalizing neurotrophic factor expression and reducing cell loss. This protection may be achieved by modulating microglial cell reactivity and increasing the number of oligodendrocytes, thereby promoting myelin repair. These findings emphasize that exercise of appropriate intensity may have significant therapeutic potential for MS.

Exercise boosts the expression of neurotrophic factors, providing an effective neuroprotective mechanism in MS, especially by promoting myelin repair and neuroregeneration. Elevated levels of factors like BDNF enhance neuronal survival, neuroplasticity, and reduce MS-induced neural damage. These findings underscore the importance of exercise in neuroprotection and repair. Future research should investigate the specific roles of various neurotrophic factors in MS and develop optimized exercise protocols to maximize neuroprotective benefits.

## 5 Future directions of research

Research to understand the potential of exercise for efficacy in humans and experimental MS is challenging and of increasing interest. However, human studies face a number of difficulties, including difficulty in obtaining histopathologic markers, assessing synaptic function, and exercise-induced neuroplasticity. Furthermore, the lack of standardization of both exercise protocols and assessment scales makes it difficult to comprehensively analyze the effects of exercise on MS patients. Despite the relative simplicity of animal models, they can unify variable control, obtain consistent and rigorous results, and help understand the mechanistic effects of exercise on the neurobiology of MS.

Future studies should explore the effects of exercise training implemented before and after disease induction in mouse models of MS (e.g., EAE, CPZ, LPC) to determine the independent and potentiating effects of exercise on preventing the development of EAE and its repair effects. Advanced molecular biology techniques, such as single-cell RNA sequencing and proteomics, could be used to identify cellular pathways and molecular targets affected by exercise. Additionally, imaging technologies like two-photon microscopy and advanced MRI could provide detailed insights into the structural and functional CNS changes induced by exercise.

In addition, further discussion should be conducted on how exercise affects MS through peripheral immunomodulation and effects on inflammatory cell accumulation in the CNS. In clinical studies, standardized exercise protocols, including type, time, duration, and intensity of exercise, should be used to more accurately assess the potential therapeutic effects of exercise on MS. Furthermore, identifying reliable biomarkers to assess the effects of exercise is essential. This could include using specific MRI parameters to track changes in brain structure and function, as well as measuring cytokine levels in serum and cerebrospinal fluid and analyzing immune cell subpopulations to evaluate systemic immune responses.

Despite a deeper understanding of the pathophysiology of MS and a growing number of therapeutic approaches to prevent MS recurrence, strategies to halt and reverse disease progression remain underdeveloped. In line with current trends in the medical field, we envision that future clinical benefits will derive directly from therapeutic decisions based on biomarkers.

## 6 Conclusion

This review analyzed animal model studies to summarize current research on how exercise mitigates MS and its underlying mechanisms. The findings indicate that exercise activates neuroprotective pathways, including attenuating pro-inflammatory/neurodegenerative microglia, stabilizing the BBB, and enhancing neurotrophin expression, all of which can reduce the neurodegenerative phenotype of MS. Future research should further explore these mechanisms in human studies, promoting the integration of exercise into comprehensive MS rehabilitation programs. A deeper understanding of these mechanisms will facilitate the development of more effective preventive strategies and personalized rehabilitation pathways for MS patients. Continued interdisciplinary research efforts are crucial to reducing the global public health burden of MS.
